# Changes in the raffinose family oligosaccharides content in the lentil and common bean seeds during malting and mashing processes

**DOI:** 10.1038/s41598-022-22943-1

**Published:** 2022-10-26

**Authors:** Alan Gasiński, Joanna Kawa-Rygielska, Dawid Mikulski, Grzegorz Kłosowski

**Affiliations:** 1 Department of Fermentation and Cereals Technology, Faculty of Biotechnology and Food Science, Wrocław University of Environmental and Life Science, Chełmońskiego 37 Street, 51-630 Wrocław, Poland; 2 Department of Biotechnology, Kazimierz Wielki University, K. J. Poniatowskiego 12 Street, 85-671 Bydgoszcz, Poland

**Keywords:** Nutrition, Plant sciences

## Abstract

Raffinose family oligosaccharides (RFOs) are sugars, which are considered anti-nutritional substances, which are not digestible by human gastric enzymes and can lead to flatulence. Legume seeds are often rich in these compounds, which can be cumbersome for many people, such as vegetarians or the population of developing countries, whose diets consists of large amounts of these food products. In this study, simple procedures used around the world in the brewing industry (malting and mashing) were used to determine, whether these processes could be applied to popular legume seeds (lentil and bean) to reduce the RFOs content. Acquired malts and worts were characterised by radically decreased concentration (up to 90%) of most ubiquitous RFOs, such as raffinose and stachyose.

## Introduction

Seeds of beans and lentils are important food product consumed worldwide. However, despite possessing high concentration of starch and proteins, the seeds possess vast array of so called anti-nutritional factors, such as phytic acid, trypsin inhibitors, tannins which diminish nutritive quality of these food products^1,2^. One of very interesting groups of anti-nutritional substances, present in the legume seeds, are so called raffinose-family oligosaccharides (RFOs)^3^. Raffinose, stachyose and verbascose are main carbohydrates from this group. The human digestive system does not produce α-galactosidase, the enzyme necessary to digest these sugars, so they are not hydrolysed and absorbed. However, in human gut microbiome there are various microorganisms capable of digesting RFOs. Unfortunately, the fermentation of RFOs can result in production of carbon dioxide, hydrogen and methane, which are components causing flatulence^4^. There are many strategies used for the improvement of nutritional quality of lentils and beans, which have proven to be successful in decreasing concentration of RFOs, such as dehulling, germination, soaking (in alcohol or water), various heat-treatments (boiling, autoclaving, microwave cooking, extrusion), enzymatic treatment, irradiation or fermentation^5,6^. However, in the scientific literature there is no mention of using malting technology to lower concentration of RFOs in legume seeds. Malting is a traditional process of seed washing, soaking, germination and drying, typically used to modify seeds of cereals, primarily barley^7^. However, as the malting procedure comprises of basic metabolic pathways and uses simple technological processes, it could be, in theory, applied basically to almost any kind of seed. Legumes can be malted, albeit with poor technological properties, as presented in the last study by Gasiński, Kawa-Rygielska, Błażewicz, Śniegowska & Zarzecki (2021)^8^. In this work, various common beans and lentils of different testa (seed coat) colour were malted and mashed, to determine, whether these procedures can be used to reduce concentration of RFOs in popularly eaten legume seeds. If malted beans and lentils would possess lower concentration of these sugars than their unmalted counterpart, they could prove as a useful substrate for production of various novel food products, such as legume-based beer, cereals, cookies or high-protein vegetarian meat substitutes or milk alternatives. Additionally, as the proteins from various legumes can be consumed by people suffering from celiac disease, non-celiac gluten sensitivity and other disorders, legume malts could be used to produce food products available for the consumption for population suffering from above-mentioned diseases^9^.

## Results

### Concentration of RFOs in legume seeds and malts

The concentration of raffinose and stachyose was assessed in the seeds of three lentil varieties and two bean varieties with different testa colours (black, brown and green lentil varieties and white and red bean varieties as well as in malts prepared from these seeds (germinated by 4, 5 and 6 day period in the case of lentil and germinated by 5 and 6 day period for the bean varieties). Results are shown in Tables 1 and 2.

**Table 1:** Concentration of stachyose and raffinose in lentil seeds and lentil malts.

Sample	Raffinose concentration [% d.w.]	Stachyose concentration [% d.w.]
BL	12.98 ± 0.66*	7.32 ± 0.27*
BL4	0.61 ± 0.11c, A	0.43 ± 0.07b, A
BL5	0.61 ± 0.06c, A	0.37 ± 0.04b, B
BL6	0.46 ± 0.04c, B	0.36 ± 0.05b, C
BR	6.77 ± 0.08***	2.88 ± 0.26***
BR4	1.63 ± 0.05a, A	0.73 ± 0.01a, A
BR5	1.62 ± 0.03a, A	0.72 ± 0.08a, B
BR6	1.35 ± 0.06a, B	0.60 ± 0.02a, C
GR	7.82 ± 0.69**	5.66 ± 0.92**
GR4	0.72 ± 0.05b, A	0.33 ± 0.03c, A
GR5	0.63 ± 0.05b, A	0.31 ± 0.02c, B
GR6	0.61 ± 0.02b, B	0.28 ± 0.03c, C

Data are shown as means ± standard deviation (n = 4). Various small letters (a, b, c) indicate homogenous groups according to the variable ‘variety’, various capital (A, B, C) letters indicate homogenous groups between malts according to the variable ‘days of germination’, various number of asterisks indicate homogenous groups between unmalted seeds according to the variable ‘variety’ (Bonferroni test, α = 0.05).

*BL* black lentil, *BL4* malt from black lentil germinated 4 days, *BL5* malt from black lentil germinated 5 days, *BL6* malt from black lentil germinated 6 days, *BR* brown lentil, *BR4* malt from brown lentil germinated 4 days, *BR5* malt from brown lentil germinated 5 days, *BRL6* malt from brown lentil germinated 6 days, *GR* green lentil, *GR4* malt from green lentil germinated 4 days, *GR5* malt from green lentil germinated 5 days, *GR6* malt from green lentil germinated 6 days.

**Table 2:** Concentration of stachyose and raffinose in bean seeds and bean malts.

Sample	Raffinose concentration [% d.w.]	Stachyose concentration [% d.w.]
WB	3.03 ± 0.33*	2.15 ± 0.20*
WB5	0.79 ± 0.03b, A	0.64 ± 0.03b, A
WB6	0.66 ± 0.05b, B	0.45 ± 0.02b, B
RB	2.20 ± 0.03**	2.45 ± 0.02**
RB5	1.45 ± 0.03a, A	1.60 ± 0.08a, A
RB6	0.69 ± 0.12a, B	0.90 ± 0.10a, B

Data are shown as means ± standard deviation (n = 4). Various small letters (a, b) indicate homogenous groups according to the variable ‘variety’, various capital (A, B) letters indicate homogenous groups between malts according to the variable ‘days of germination’, various number of asterisks indicate homogenous groups between unmalted seeds according to the variable ‘variety’ (Bonferroni test, α = 0.05).

*WB* white beans, *WB5* malt from white bean germinated 5 days, *WB6* malt from white bean germinated 6 days, *RB* red beans, *RB5* malt from red bean germinated 5 days, *RB6* malt from red bean germinated 6 days.

Verbascose was not detected in any of the analysed types of seeds, however, this result is not very surprising, as this sugar is often found in faba beans, lupins, mung beans but not as abundant in common beans and lentils^10^. Stachyose and raffinose was detected in all the legume seeds and legume malt samples, but concentration of these sugars varied significantly. Raffinose was detected in greater quantity than stachyose in all unmalted seeds samples, with the exception of RB, and its content was in the range from 2.20% (d.w.) (RB) to 12.98% (d.w.) (BL). The lowest stachyose concentration for unmalted seeds was detected in WB (2.15% d.w.) and the greatest in BL (7.32% d.w.). Bean samples were characterised with lower concentration of raffinose, stachyose as well as total RFO content than lentil seeds.

### Concentration of RFOs in worts produced from the lentil malts

Concentration of raffinose and stachyose in wort samples acquired during the congress mashing of the lentil malts is shown in Table 3. Worts were acquired from malts produced from three different lentil varieties with various testa colours (black, brown and green), which were germinated by different time periods (4, 5 and 6 days). Worts could not have been acquired from the bean malts due to the filtration problems during congress mashing procedure.

**Table 3:** Extract content and concentration of stachyose and raffinose in worts produced from lentil malts.

Sample	Extract content [% w/w]	Raffinose concentration [g/dm^3^]	Stachyose concentration [g/dm^3^]
BL4W	4.75 ± 0.07a, C	0.18 ± 0.02c, A	n.d
BL5W	4.95 ± 0.02a, B	0.16 ± 0.02c, B	n.d
BL6W	5.00a, A	0.12 ± 0.01c, B	n.d
BR4W	3.40b, C	0.60 ± 0.16b, A	0.23 ± 0.03b, A
BR5W	3.50b, B	0.48 ± 0.10b, B	0.13 ± 0.02b, B
BR6W	3.60b, A	0.47 ± 0.16b, B	0.14 ± 0.04b, B
GR4W	4.80a, C	0.70 ± 0.04a, A	0.32 ± 0.03a, A
GR5W	4.90a, B	0.64 ± 0.04a, B	0.29 ± 0.03a, B
GR6W	5.00a, A	0.60 ± 0.02a, B	0.31 ± 0.02a, B

Data are shown as means ± standard deviation (n = 6). Various small letters (a, b, c) indicate homogenous groups according to the variable ‘variety’, various capital (A, B) letters indicate homogenous groups between malts according to the variable ‘days of germination’ (Bonferroni test, α = 0.05). N.d. stands for “not detected”.

*BL4W* wort from black lentil malt germinated 4 days, *BL5W* wort from black lentil malt germinated 5 days, *BL6W* wort from black lentil malt germinated 6 days, *BR4W* wort from brown lentil malt germinated 4 days, *BR5W* wort from brown lentil malt germinated 5 days, *BRW6* wort from brown lentil malt germinated 6 days, *GR4W* wort from green lentil malt germinated 4 days, *GR5W* wort from green lentil malt germinated 5 days, *GR6W* wort from green lentil malt germinated 6 days.

Acquired lentil worts were characterised with lower concentration of stachyose than raffinose. Average concentration of stachyose in lentil worts was equal to 0.16 g per dm^3^ of wort and average concentration of raffinose in lentil worts was almost three times higher, equal to 0.44 g per dm^3^ of wort. Worts acquired from black lentil malts were characterised with the lowest concentration of raffinose (0.15 g/dm^3^) and stachyose (stachyose was absent in these worts). Average concentration of stachyose in worts from brown lentil malts was equal to 0.17 g/dm^3^ and in worts from green lentil malt it was almost two times higher (0.31 g/dm^3^). Lower difference can be noted for the content of raffinose in these worts: brown lentil worts were characterised with raffinose concentration of 0.52 g/dm^3^ and green lentil worts with raffinose concentration of 0.65 g/dm^3^.

## Discussion

### Concentration of RFOs in legume seeds and malts

Analysis of the acquired data shows, that different varieties of the same species have significantly different concentration of the raffinose and stachyose. Malts prepared from the legume seeds were characterised with drastically lower concentration of raffinose and stachyose than their unmalted counterparts. RFOs are abundant in the plant kingdom synthesised and utilised many plants^10^. They are accumulated in various parts of the plants, such as seeds or leaves in many different species, such as various legumes, grains sich as maize or rice and in tissues of various other plants, as, for example, cucumbers or coleuses^11–15^. Previous study by Gangl & Tenhaken (2016)^16^ conducted on model plant seeds (*Arabidopsis thaliana*) have shown, that raffinose was required for the fast germination of the seeds in the dark. The drastic decrease of the raffinose content in lentil seeds would suggest that *Lens culinaris* and *Phaseolus vulgaris* also uses raffinose and stachyose during the germination. However, soybean seed research results^17^ suggested, that these sugars are not required for the germination, albeit, when present, they are used by the germinating seed embryo. Data acquired in this study have shown that malting procedure (combination of seed hydration, germination and then kilning) has significant influence on the RFO content of legume seeds. Average concentration of stachyose in black lentil malts was 20 times lower thanthe stachyose content of BL and average concentration of raffinose in black lentil malts was 23 times lower than raffinose content of BL. Similar decrease could be noted for the stachyose content in the green lentil malt (decrease in malts equal to the 5.4% of the stachyose content in GR), but a lower change was found for the raffinose concentration in green lentil malts (raffinose content in green lentil malts was equal to 8.4% the content in GR). Brown lentil malts were characterised with the highest concentration of raffinose and stachyose. Additionally, malting had the least significant impact on the concentration of raffinose and stachyose in the BR4, BR5 and BR6. It might indicate that seeds which accumulate higher concentrations of RFOs are more capable of their utilisation, which is logical, reasonable and found in many instances in plant kingdom^18,19^. Malting decreased average concentration of raffinose in brown lentil malts to 22.6% of the starting value and decreased stachyose concentration to 23.7% of the concentration found in BR. Analysed beans were characterised with lower concentration of RFOs than lentils, but malting procedure performed on this seeds resulted in lower decrease of raffinose and stachyose. Malting decreased stachyose concentration in white bean malts to 25.3% of the starting value and reduced stachyose concentration in red bean malts to 51% of the amount present in the RB sample. Decrease in the raffinose concentration was similar. Malting of the white beans resulted in the decrease of raffinose to 23.9% of the concentration in WB and, in the case of red beans, to 48.6% of the concentration present in RB. Not only a seed species and variety should be mentioned as a factor influencing total concentration of RFOs. The length of the germination during the malting period also significantly affected content of RFOs in legume malts. Typical barley malts are germinated for 5 days, but some malthouses possess capacity to malt seeds for additional day (however, it increases losses for the malthouse). Malting for four days is also a viable option, if the maltsters want to speed up the production process, but the produced malt is of inferior quality compared to the malt germinated for the full length of the process. Malts germinated 1–3 days (called ‘short’ or ‘chit’ malts) are characterised with not adequate technological parameters and are not typically produced in the larger scale^7,20^. These are the reason why the most viable period of germination time was tested. Analysis of malts show, that 6-day germination time during malting had greatest influence on the concentration of raffinose and stachyose in lentil malts as well as bean malts. It decreased, on the average, concentration of stachyose present in the unmalted seeds by 90% and concentration of raffinose by 89.5%, in comparison to 4-day germination time which decreased concentration of stachyose present in the unmalted seeds by 87.5% and concentration of raffinose by 87.3%. Similar phenomenon can be seen in the bean malts, but the discrepancies between 5-day germination time and 6-day germination time were greater. 5 days of germination in the malting period resulted in the loss of 54% of raffinose and 52.4% loss of stachyose, while 6-day germination time resulted in the loss of 73.5% of raffinose and 71% of stachyose. This data shows, that if the goal of malting would be to produce substrate low in RFOs, in the case of lentil the germination time could be as short as 4 days, but to acquire acceptable results in reduction of RFOs in beans, 6 days of germination or more would be more recommended.

### Concentration of RFOs in worts produced from the lentil malts

Mashing in the brewing process is a procedure, which is used to hydrolyse various substances present in the malts with the use of endogenous enzymes. Main goal of the mashing is complete hydrolysation of starch and adequate hydrolysation of proteins, which is why most of the research analysing malt enzymatic potential concentrates mainly on amylolytic enzymes (α-amylases, β-amylases and glucoamylases) and various proteases^21,22^. Additionally, typically malted seeds do not possess high concentration of RFOs. This is why in the literature there is not much data about activity of enzymes capable of hydrolysing RFOs in various malts (such as legume α-galactosidases), as well as no data about concentration of various RFOs in wort samples^23^. Different germination time during the malting process also had an impact on the amount of raffinose and stachyose present in the worts. Worts produced from malts germinated 4 days were characterised with higher concentration of raffinose (0.49 g/dm^3^) and stachyose (0.18 g/dm^3^) than malts germinated 5 and 6 days, in which raffinose concentration ranged from 0.43 to 0.40 g/dm^3^ and stachyose concentration ranged from 0.15 to 0.14 g/dm^3^. However, it can be seen that differences are not as crucial as could be seen in malts prior to the mashing, therefore, if the goal of the technological procedure would be to produce worts from lentil malts with low concentration of RFOs, 4-day germination time might prove adequate, decreasing production time and costs.

## Materials and methods

### Raw material

Raw material used in this study were seeds of three lentil (*Lens culinaris*) varieties and two common bean (*Phaseolus vulgaris*) varieties. Each variety was of different colour: black lentil of Beluga variety (BL), brown lentil of Firat 87 variety (BR), green lentil of variety Eston (GR), white bean of variety Piękny Jaś (WB) and red bean of variety Kreacja (RB). These are varieties (with the exception of Beluga lentil) commonly grown in the region. Black lentil of Beluga variety is the only one black lentil variety grown commercially in the area. Lentil and bean seeds were acquired from BioPlanet Company (Leszno near Warsaw, Poland). All procedures concerning use of the plant seeds were conducted in accordance to the institutional, national and international guidelines and legislation.

### Chemicals and analytical standards

Analytical standards used for this study were raffinose, verbascose and stachyose, HPLC grade (99% purity) (Sigma Aldrich, Saint Louis, MO, USA). Additional reagents used in this study were sulphuric acid (H_2_SO_4_, 99.999% purity, Sigma Aldrich, Saint Louis, MO, USA) and glucoamylase: Saczyme Plus enzyme preparation from Novozymes (Denmark) with the activity of 750 AGU/g (one unit of amyloglucosidase activity is defined as the amount of enzyme required to release one µmole of D-glucose reducing-sugar equivalents per minute from soluble starch at pH 4.5 and 40 °C).

### Malting procedure

Malting was performed in different conditions for lentil seeds and common bean seeds, because there are significant physiological differences between these species. Malting procedure consisted of seed steeping (hydration), seed germination and seed kilning (drying).

#### Seeds cleaning and weighing

Before seeds were weighed, the lentils and beans were manually assessed and damaged seeds as well as seeds with visible discolouration were discarded. After cleaning and sorting out, moisture of the seeds was assessed using automatic NIR grain analyser Infratec 1241 (Foss, Hilleroed, Denmark) equipped with program for analysis of legume seeds to determine the starting moisture content of seeds. Seventy gram portions of lentils and seventy gram portions of beans were weighed to the corresponding stainless steel perforated containers (12 containers for each seeds type). The weight of the empty containers, weighed seeds and containers with seeds were carefully noted. These measurements were used in the future to determine the changing moisture content of the steeped seeds. The perforated stainless steel container with the seeds inside will from now on be mentioned as the ‘malting kit’.

#### Seeds steeping

Steeping was conducted in the water–air cycle, using 50dm^3^ stainless steel chamber and KK 240 Smart Pro germination chamber (Pol-Eko Aparatura, Wodzisław Śląski, Poland). Malting kits with lentil seeds were submerged in the tap water for 7 h (water temperature was equal to 18 °C), then kept in germination chamber (18 °C, relative humidity 90%) for 18.5 h. After 18.5 h, lentil seeds were submerged another time for 5 h (water temperature 18 °C) and then kept in the germination chamber for 17.5 h (18 °C, relative humidity 90%). Malting kits with beans were submerged in the tap water for 8 h (water temperature was equal to 20 °C), then kept in germination chamber (20 °C, relative humidity 90%) for 16 h. After 16 h, malting kits with beans were submerged another time for 6 h (water temperature 20 °C) and then kept in the germination chamber for 18 h (20 °C, relative humidity 90%). After each step of the seed steeping (after first submerging, after first air rest in the humid atmosphere, after second submerging and after the second air rest in the humid atmosphere), the malting kits were weighed and the moisture content of the seeds were calculated based on the changed weight of the malting kit. It was assumed that weight increase was equal to the amount of water absorbed by the seeds. After the steeping process, malting kits were weighed kept in the germination chamber for the seed germination. The moisture content of the bean seeds at the end of the steeping process was in the range of 57–59%, while the moisture content of lentil seeds was in the range of 58–60%.

#### Seeds germination

Lentil seeds were germinated at 18 °C and bean seeds were germinated at 20 °C. Each day, the malting kits were weighed and the weight loss was supplemented by spraying the seeds with sterile, distilled water. Each day the seeds were visually assessed to determine the level of acrospire growth and were manually mixed to avoid root entanglement. After 96 h of germination, 4 malting kits with each lentil type (black, brown and green) were removed from the germination chamber and kilned (as described in the Sect. “Seeds drying”). After 120 h of germination, four malting kits with each lentil type and six malting kits of each bean type (red and white) were removed from germination chamber and kilned. After 144 h of germination, last four malting kits with each lentil type and last six malting kits with each bean type were removed from germination chamber and kilned. The beans were dried only after the 120 h and 144 h, because the acrospire growth after 96 h was unsatisfactory.

#### Seeds drying

After the germination process, each batch of malting kits was dried in the UF110 Plus dryer (Memmert GmbH + Co, Schwabach, Germany) at the following conditions: 50 °C (17 h and 50 min), ramp up to 65 °C (10 min), 65 °C (2 h and 50 min), ramp up to 82 °C (10 min), 82 °C (2 h). The malts, after the drying process, malts of one type from different malting kits were mixed together and transferred to tightly closed containers, to prevent moisture absorption during cooling period. The germination and drying processes allowed for the production of 15 different malt types:

- black lentil malt germinated for 96 h (BL4);
- black lentil malt germinated for 120 h (BL5);
- black lentil malt germinated for 144 h (BL6);
- brown lentil malt germinated for 96 h (BR4);
- brown lentil malt germinated for 120 h (BR5);
- brown lentil malt germinated for 144 h (BR6);
- green lentil malt germinated for 96 h (GR4);
- green lentil malt germinated for 120 h (GR5);
- green lentil malt germinated for 144 h (GR6);
- red bean malt germinated for 120 h (RB5);
- red bean malt germinated for 144 h (RB6);
- white bean malt germinated for 120 h (WB5);
- white bean malt germinated for 144 h (WB6);

Before the mashing procedure (Sect. “Congress mashing of the legume malts”) malts were ground with the use of Bühler Miag disc mill DLFU (Bühler, Uzwil, Switzerland), according to the Analytica EBC 4.5.1 method^24^. Malts and unmalted seeds before analysis of RFOs concentration were ground using IKA A10 basic mill (Staufen, Germany).

### Congress mashing of the legume malts

Congress worts were produced in the automated laboratory mashing machine (LB Electronic, Lochner Labor and Technik, Berching, Germany) according to the Analytica EBC method 4.5.1. Wort, after filtration, was collected for analyses. Congress worts from legume malts were prepared in duplicate. It was not possible to produce congress worts from the bean malts, because the first 100cm^3^ of the wort, which had to be reversed into the funnel to start the filtration process could not have been acquired. Congress mashing, therefore, allowed for production of 9 congress wort samples:

- wort from black lentil malt germinated for 96 h (BL4W);
- wort from black lentil malt germinated for 120 h (BL5W);
- wort from black lentil malt germinated for 144 h (BL6W);
- wort from brown lentil malt germinated for 96 h (BR4W);
- wort from brown lentil malt germinated for 120 h (BR5W);
- wort from brown lentil malt germinated for 144 h (BR6W);
- wort from green lentil malt germinated for 96 h (GR4W);
- wort from green lentil malt germinated for 120 h (GR5W);
- wort from green lentil malt germinated for 144 h (GR6W);

Extract content of the legume malt worts was analysed with the use of densimeter (DMA 35, Anton Paar, Graz, Austria). For each wort sample, measurement was performed in triplicate (which equals six extract measurements per one type of malt).

### Analysis of the RFO content in the legume seeds, legume malts and worts produced from legume malts

#### Extraction and enzymatic hydrolysis of maltotetraose, maltotriose and maltose in the legume seeds and malts

To carry out necessary identification and quantification of raffinose, stachyose and verbascose, various disaccharides, trisaccharides and tetrasaccharides (products of starch hydrolysis, such as maltose, matotriose and maltotetraose) had to be hydrolysed, because otherwise these sugars would have prevented optimal identification and quantification of RFOs. Two grams of ground seed or malt sample and 20cm^3^ H_2_SO_4_ (5 mmol concentration) were added to the Erlenmeyer flask. The flasks were shaken for 3 h at the room temperature. After 3 h, the sulphuric acid solution was filtered through syringe filter (pore size 0.45 µm). 1cm^3^ of the filtered solution was added to test tube. Glucoamylase preparation (10mm^3^) was added to the test tube, which was then closed with cap and incubated for 1 h at temperature of 60 °C. After the incubation, the solution was centrifuged (10,000 rpm, 5 min) and supernatant was added to the chromatographic vial and analysed.

#### Preparation of worts produced out of legume malts for the chromatographic analysis

The wort (1cm^3^), after the filtration through the paper funnel (in accordance to the Analytica 4.5.1 method) was pippeted to the test tube and diluted 10 times using H_2_SO_4_ (5 mmol). Glucoamylase preparation (10mm^3^) was added to the test tube, which was then closed with cap and incubated for 1 h at temperature of 60 °C. After incubation, worts were centrifuged and supernatant was added to the chromatographic vials and analysed (Supplementary Fig. 1).

#### Chromatographic analysis of the RFOs

Concentration of RFOs (raffinose, stachyose and verbascose) was assessed using high performance liquid chromatography (HPLC). Chromatographic analyses were performed using Agilent Technologies model 1220 (Agilent Technologies, Santa Clara, CA, USA) apparatus equipped with a thermostat and refractometric detector (RID). The separation was performed using a Hi-Plex H (Agilent Technologies®, Santa Clara, CA, USA) column. The mobile phase was 5 mmol H_2_SO_4_ flowing at 0.6cm^3^/min under isocratic conditions. The working temperature of the column was 60 °C. The working temperature of the refractometric detector was 50 °C. External standards (ESTD) and the appropriate calibration curves were used to determine the concentration of the analysed compounds. The separation parameters were in accordance with the recommendations of the chromatography column manufacturer. Limit of detection (LOD) for raffinose was equal to 0.011 g per dm^3^ of the solution (extract or wort); limit of quantitation for raffinose was equal to 0.032 g per dm^3^ of the solution. Limit of detection (LOD) for stachyose was equal to 0.005 g per dm^3^ of the solution; limit of quantitation for stachyose was equal to 0.015 g per dm^3^ of the solution. Limit of detection (LOD) for verbascose was equal to 0.021 g per dm^3^ of the solution; limit of quantitation for stachyose was equal to 0.064 g per dm^3^ of the solution. Linearity equations were as follows: for raffinose y = 76810x − 817.4 (R^2^ = 0.9997); for stachyose y = 208,455.0x + 193.34 (R^2^ = 0.9999); for verbascose y = 331911x + 2520.9 (R^2^ = 0.9987). Sample chromatograms of RFOs content in lentil and bean malts and unmalted seeds are provided in the Supplementary Material (Supplementary Fig.1–5).

### Data analysis

The results of the analysis of oligosaccharide content of seeds and malts were statistically analysed using the SPSS Statistics 26 program from IBM (Armonk, NY, USA). Two-way ANOVA was used for the determination of differences in concentration of RFOs between lentil malts as well as bean malts (with the variables: length of germination and seed variety). One-way ANOVA was used for the determination of differences between malted seeds and unmalted seeds (with the variable of seed variety). Two-way ANOVA for the analyses of lentil worts (with the variables: days of germination, lentil variety). Bonferroni test was used as a post-hoc test with α < 0.05. Extraction and enzymatic hydrolysis was performed in duplicate for each of the malts and seeds and each of the extracts was analysed chromatographically in two repetitions, which resulted in four readings per type of seed/malt. Worts were prepared in duplicate during the congress mashing procedure. Each hydrolysed wort was analysed chromatographically in three repetitions, which resulted in six reading per type of wort. Extract content of the worts was analysed in triplicate, which resulted in six readings per type of wort.

## Conclusions

Lentil seeds and bean seeds are rich in raffinose and stachyose, but conducted study shows, that malting procedure can be successfully used as a tool to decrease concentration of these sugars. Malting with 6-day germination time radically decreased raffinose and stachyose content in lentil seeds. The reduction in the RFO content was smaller in the case of bean malts, but these seeds were characterised with lower concentration of RFOs than lentil seeds. Mashing process allowed producing lentil worts with very low concentration of stachyose (ranging from 0.13 g/dm^3^ to 0.32 g/dm^3^) and raffinose (ranging from 0.40 g/dm^3^ to 0.49 g/dm^3^). These results show, that malting and mashing procedures can be used to modify legume seeds and produce novel raw material adequate for production of various food products with reduced RFOs content.

## Supplementary Information

Supplementary Figures.

## Data Availability

The datasets used and analysed during the current study are available from the corresponding author on reasonable request.
