# Supplementary material for: Changes in the raffinose family oligosaccharides content in the lentil and common bean seeds during malting and mashing processes
Source: Sci Rep. 2022 Oct 26;12:17911. doi: 10.1038/s41598-022-22943-1 (PMC9606247; doi:10.1038/s41598-022-22943-1)
Supplement: Supplementary file 1 — Supplementary Figures. [file 41598_2022_22943_MOESM1_ESM.docx]

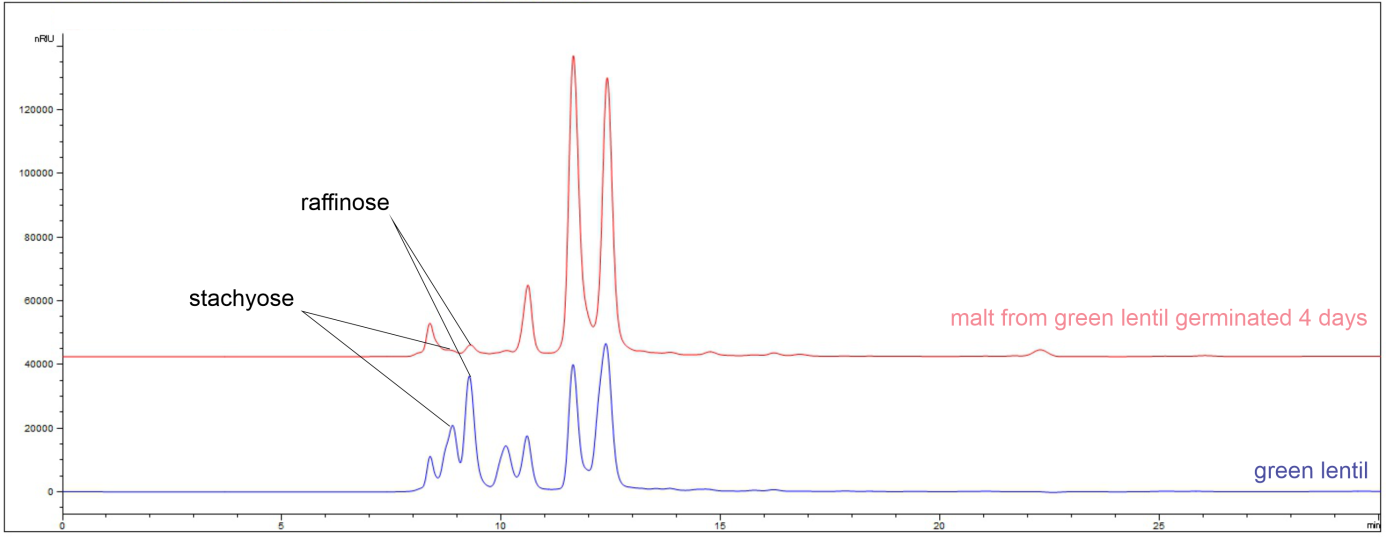


Figure 1. HPLC chromatogram of extract from green lentil and green lentil malt germinated for 4 days.


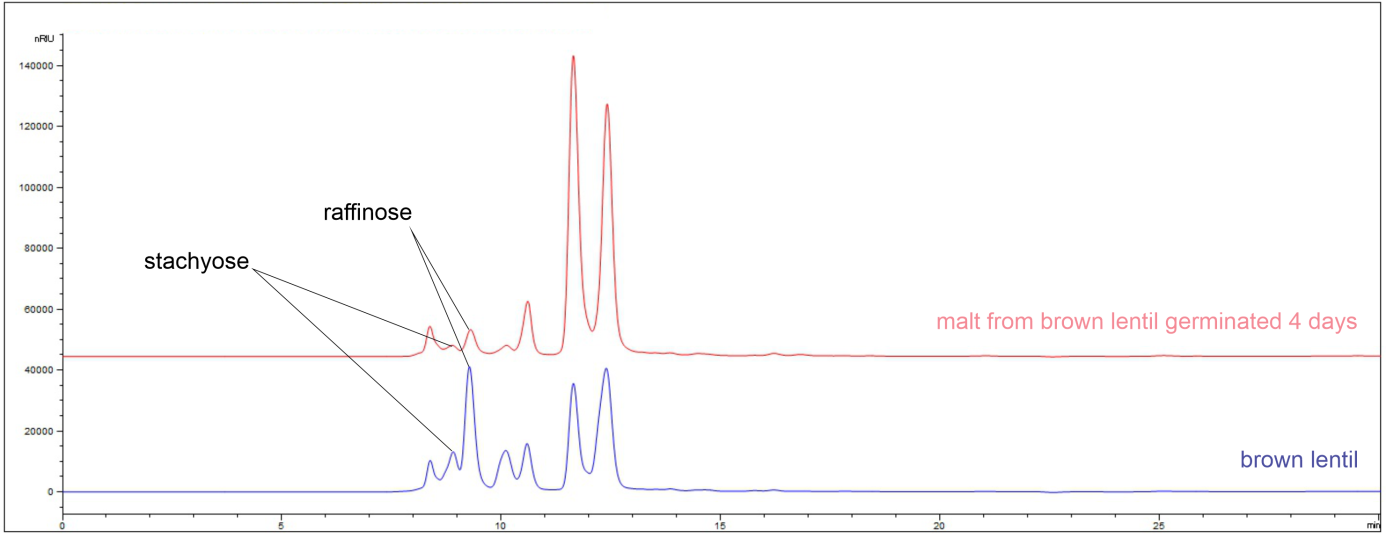


Figure 2. HPLC chromatogram of extract from brown lentil and brown lentil malt germinated for 4 days.


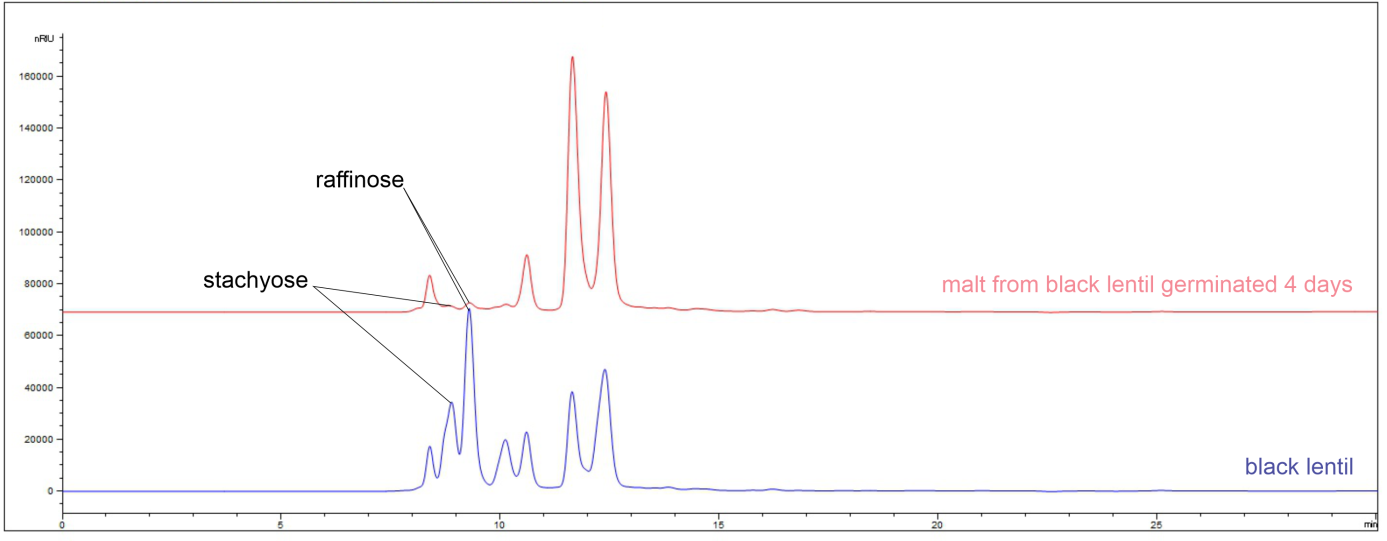


Figure 3. HPLC chromatogram of extract from black lentil and black lentil malt germinated for 4 days.


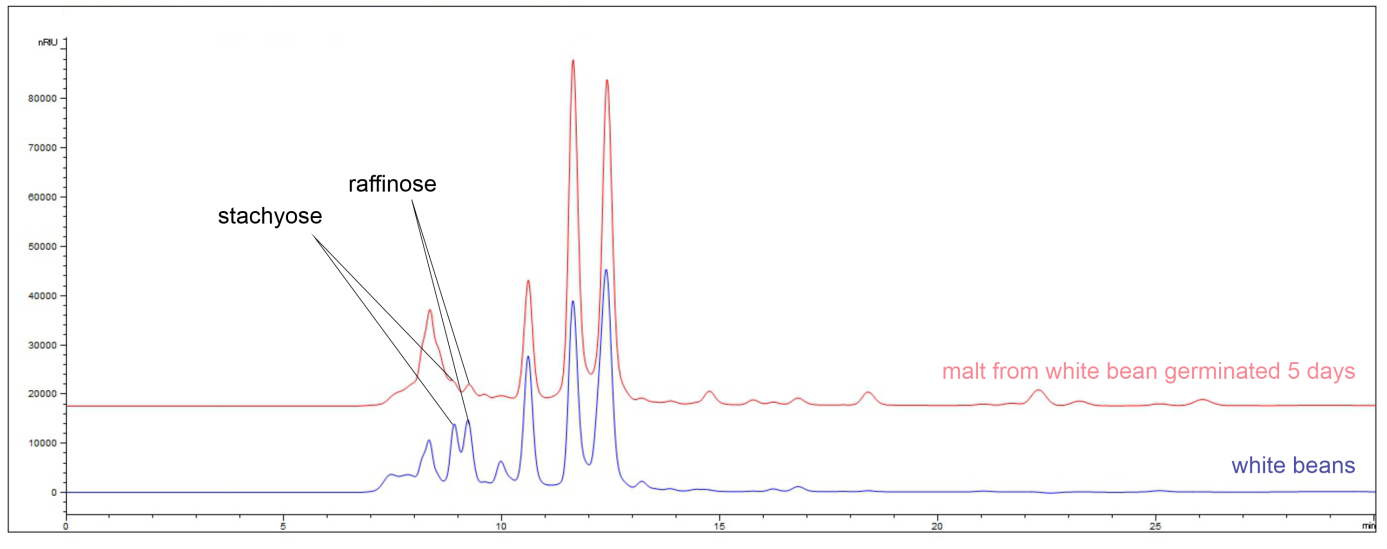


Figure 4. HPLC chromatogram of extract from white bean and white bean malt germinated for 5 days.


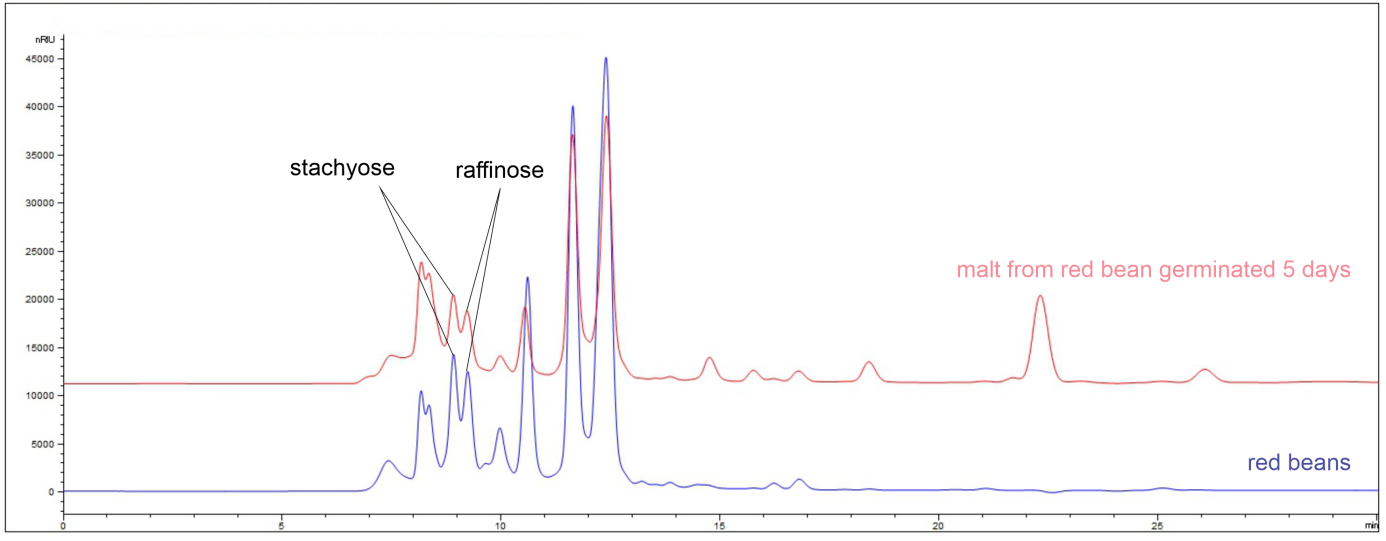


Figure 5. HPLC chromatogram of extract from red bean and red bean malt germinated for 5 days.ss
